# *Macrophage migration inhibitory factor* gene rs755622 G/C polymorphism and coronary artery disease: A meta-analysis of 8,488 participants

**DOI:** 10.3389/fcvm.2022.959028

**Published:** 2022-09-14

**Authors:** Yan-yan Li, Hui Wang, Yang-yang Zhang

**Affiliations:** ^1^Clinical Research Center, The First Affiliated Hospital of Nanjing Medical University, Nanjing, China; ^2^Department of Geriatrics, The First Affiliated Hospital of Nanjing Medical University, Nanjing, China; ^3^Department of Cardiology, The First Affiliated Hospital of Nanjing Medical University, Nanjing, China; ^4^Department of General Practice, The First Affiliated Hospital of Nanjing Medical University, Nanjing, China

**Keywords:** *MIF*, rs755622, polymorphism, coronary artery disease, genetic, *macrophage migration inhibitory factor*

## Abstract

**Background and aims:**

*Macrophage migration inhibitory factor* (*MIF*) gene rs755622 G/C polymorphism was suggested to be associated with CAD risk. However, due to the different results among the individual studies, no agreement has been reached till now. Therefore, the meta-analysis on the association of *MIF* gene rs755622 G/C polymorphism with CAD was performed.

**Methods and results:**

The association between them was evaluated by calculating the pooled odds ratios (ORs) and the corresponding 95% confidence intervals (CIs). The random-effects models were used because of the significant heterogeneity among them. In this meta-analysis, 8,488 subjects from 9 studies were included. The *MIF* gene rs755622 G/C polymorphism was significantly associated with CAD under the allelic (OR: 1.213, 95% CI: 1.039–1.417, *P* = 0.014), recessive (OR: 1.945, 95% CI: 1.214–3.115, *P* = 0.006), dominant (OR: 0.781, 95% CI: 0.617–0.989, *P* = 0.041), homozygous (OR: 2.057, 95% CI: 1.289–3.284, *P* = 0.003), and additive (OR: 1.327, 95% CI: 1.081–1.630, *P* = 0.007) genetic models.

**Conclusion:**

*MIF* gene rs755622 G/C polymorphism was significantly related to CAD, especially in the Chinese population. Persons with the C allele of the *MIF* gene rs755622 G/C polymorphism might be susceptible to CAD.

## Introduction

Coronary artery disease (CAD) is a serious disease that endangers human health and life. In developed Western countries, such as Europe and the United States, CAD has decreased incidence compared with 30 years ago but remains the main cause of death in the population (1). In China, with the improvement of people’s living standards, changes in diet structure and eating habits, and increased social pressure, the incidence and mortality of CAD increase. At present, the number of cardiovascular diseases in China has reached 330 million. In 2018, the crude mortality rate of CAD was 120.18/100,000 in urban areas and 128.24/100,000 in rural areas. The CAD has become a heavy burden on Chinese individuals and society (2).

The pathogenesis of CAD is the atherosclerosis of the coronary artery. Risk factors for CAD include smoking, hyperlipidemia, hypertension, and diabetes. Studies found that CAD is also a chronic inflammatory disease, and a variety of inflammatory factors play important roles in the occurrence and development of CAD. The macrophage migration inhibitor (MIF) is a homologous trimer protein that can locally inhibit macrophage migration and promote the inflammatory response. MIF can be expressed in many cells and tissues, including mononuclear phagocytes, vascular smooth muscle cells, and cardiomyocytes (3). MIF is an autocrine factor of macrophages, which plays an important role in inflammation and is a key mediator of arteriosclerosis (4).

The human *MIF* gene, located in 21q11.2, spans about 2,119 bp, contains 3 exons and 2 introns, and encodes 115 amino acids. The *MIF* gene –173G/C polymorphism (rs755622) is located in the promoter, which is located at the binding site of the response element of transcription factor activator protein 4 (AP4). This rs755622 polymorphism may affect the affinity of the response element of AP4, thus affecting the transcription level of the *MIF* gene (5). The *MIF* gene polymorphism and increased MIF protein concentration are associated with the incidence of ulcerative colitis, psoriasis, and tuberculosis. These diseases are often accompanied by inflammatory reactions, which illustrate the key role of MIF in promoting the occurrence and development of these diseases (3).

Many studies suggested that the *MIF* gene –173 G/C polymorphism is associated with the risk of CAD, but some studies confirmed no association between *MIF* gene –173 G/C polymorphism and CAD. Ji et al. found that patients with CAD have higher frequencies of C and –173CC genotypes than the control. The *MIF* gene rs755622 polymorphism is suggested to be related to CAD in a Chinese population, and the plasma MIF level is a predictor of plaque stability (6). Similarly, Fahriye et al. found that the low frequency of the GG genotype and G allele of the *MIF* gene –173 G/C polymorphism may be associated with the etiopathogenesis of patients with CAD in a Turkish population (7). Nevertheless, Tereshchenko et al. found no significant difference in the distribution of *MIF* gene –173G/C genotypes between CAD and control groups in Czech or Russian populations (8). Additionally, Wu et al. reported that the –173G/C polymorphism of the *MIF* gene does not contribute to CAD risk in another Chinese population (9).

Recently, similar meta-analyses on the association of *MIF* gene –173G/C polymorphism and CAD have been published. Li et al. carried out a meta-analysis to evaluate the association of *MIF* gene –173 G/C polymorphism and CAD susceptibility. Evidence of associations between them in Asian and Caucasian populations is also found. However, only six individual studies are included in their meta-analysis. Therefore, Li et al.’s meta-analysis results may not be as comprehensive as those obtained from the current meta-analysis (10). Lian et al. published a meta-analysis to investigate the relationship between them and also found that the *MIF* gene promoter –173 locus is significantly correlated with CAD risk and that the C allele is a risk factor for CAD (11). However, two studies published by a similar author group are included in their meta-analysis (12, 13). The adopted data may overlap, thus affecting the credibility of the results. Hence, Lian’s results may not be as objective as those from the current meta-analysis.

In this study, case–control studies on the correlation between *MIF* promoter region –173C/G polymorphism and CAD risk are collected, and systematic analysis is conducted to understand the association between CAD risk and *MIF* promoter region –173C/G polymorphism (Supplementary Table 1).

## Materials and methods

### Publication search and inclusion criteria

The relevant studies in the literature were searched from electronic databases such as the China National Knowledge Infrastructure, WanFang database, Weipu database, Web of Science, and PubMed. In the search process, keywords as “CAD,” “coronary heart disease,” “*macrophage migration inhibitory factor*,” “*MIF*,” “rs755622,” “gene,” “mutation,” and “polymorphism” were used. The final search was updated on 31 May 2022. The qualified studies were published between 2006 and 2018.

The included studies should conform to the subsequent criteria. The included studies should (a) evaluate the association of *MIF* gene rs755622 G/C polymorphism with CAD, and (b) the diagnosis of CAD was defined as the stenosis degree of the left main trunk, left anterior descending branch, left circumflex branch, right coronary artery, and any one of the main branches ≥ 50% by coronary angiography examination. (c) The research should be case-control or cohort studies. If multiple studies were retrieved by one author group, the data would be analyzed. If there was overlapped data, the data could be merely adopted for one time. The data with more sample size would be adopted. The studies that did not provide enough data should not be analyzed. The selected studies’ quality would be evaluated by using the Newcastle-Ottawa Scale (NOS) score.

### Data extraction

The data extraction process was carried out by three researchers following the standard procedures. Two investigators completed literature retrieval, quality evaluation, and data extraction independently. If the two investigators have differences, they will discuss them with the third investigator. However, this did not often occur and had less effect on the overall NOS score. The obtained literature was then scored according to the NOS score evaluation system. According to the scoring rules, the score of the literature was between 0 and 9 points. Two independent researchers graded strictly according to the grading guidelines. A literature score greater than or equal to 6 is considered high quality. Data that met the inclusion criteria were extracted from the literature, and data extraction tables were developed. The table includes the first author, publication year, study region, race, number of genotypes in the CAD group and control group, genotyping method, the sample size in each group, and *P*-value of Hardy-Weinberg equilibrium (HWE) in the control group. The population in the case group might be affected by disease pressure (a type of selection pressure), which would make its genotype distribution deviate from HWE. However, the genotype distribution of the control group without disease should be consistent with HWE. Therefore, in the meta-analysis of genetic association studies, it is necessary to test whether the genotype distribution of the control group in the case-control study is consistent with HWE.

### Statistical analyses

In this study, there were six genetic models, namely, allelic (C allele distribution frequency), recessive (CC vs. GG + GC), dominant (GG vs. GC + CC), heterozygous (GC vs.GG), homozygous (CC vs. GG), and additive (C allele vs. G allele). The pooled odds ratios (ORs) and corresponding 95% confidence intervals (CIs) were adopted to assess the association between *MIF* gene rs755622 G/C polymorphism and CAD.

The *P*_*heterogeneity*_ and *I*^2^ were calculated to assess the heterogeneity among the included studies (14). On the condition of *P*_*heterogeneity*_ < 0.05, the random-effects models would be utilized to analyze the data. Conversely, the data analysis would be performed by using the fixed effects models (15). *Z*-test was used to calculate the pooled ORs under the six genetic models. The open-source software R was used to perform Fisher’s exact test and evaluate the HWE (16). The publication bias was evaluated by using Begg’s test (17, 18). *P* < 0.05 was considered statistically significant difference in the above tests. All data were analyzed using Stata12.0 software.

## Results

### Studies and populations

There were nine studies in the meta-analysis. The NOS scores were greater than 6 in all the included studies, which suggested the included literature qualities were high. In total, 3,843 CAD cases and 4,645 controls were identified according to the evaluation indexes (Table 1) (3, 6–9, 12, 19–21). Of the nine studies, seven were concentrated on the Chinese population (3, 6, 9, 12, 19–21), while the other two studies were concentrated on Caucasians (7, 8).

**TABLE 1:** Characteristics of the studies of the association between the *MIF* gene rs755622 polymorphism and CAD included in the meta-analysis.

Authors	Year	Region	Ethnicity	CAD			Control			Matching criteria	Genotyping method	Sample size (CAD/control)	*P*_*HWE*_
									
				GG	GC	CC	GG	GC	CC				
Lin et al. (19)	2008	China	Chinese	41	21	5	58	12	2	Age, sex, smoking history, TG, ethnicity	PCR-RFLP	67/72	0.18
Hua et al. (6)	2015	China	Chinese	46	14	10	136	44	6	Age, sex, cigarette smoker, drinking, hypertension, HDL-C, LDL-C, TC, TG, ethnicity	PCR- sequence	70/186	0.31
Qian and Yin. (3)	2018	China	Chinese	71	26	21	142	73	14	Age, smoker, drinking, hypertension, DM, ethnicity	PCR-RFLP	118/229	0.27
Fahriye et al. (7)	2017	Turkey	Caucasian	13	16	6	58	40	2	Sex, ethnicity	PCR-RFLP	35/100	0.10
Tereshchenko et al. (8)	2009	Czech, Russia	Caucasian	327	120	12	229	73	9	Ethnicity	PCR-SSP	459/311	0.29
Xu (12)	2015	China	Chinese	1,374	889	157	1,799	1,023	137	Age, sex, BMI, ethnicity	PCR-Taqman	2,420/2,959	0.58
Shan et al. (20)	2006	China	Chinese	115	23	0	152	11	0	Age, ethnicity	PCR-RFLP	138/163	0.66
Wu et al. (9)	2014	China	Chinese	21	167	297	20	136	275	Age, BMI, Glucose, LDL-C, TC, TG, Apo (A), Apo (B), Apo (E), ethnicity	PCR-Taqman	485/431	0.5
Dai et al. (21)	2016	China	Chinese	38	10	3	115	75	4	Age, BMI, TC, TG, ethnicity	PCR-sequence	51/194	0.04*

*P < 0.05. MIF, macrophage migration inhibitory factor; CAD, coronary artery disease; CI, confidence interval; OR, odds ratio; CAD size, the total number of CAD cases; control size, the total number of control group; BMI, body mass index; TC, total cholesterol; LDL-C, low-density lipoprotein cholesterol; HDL-C, high-density lipoprotein cholesterol; TG, triglycerides; DM, diabetes mellitus; PCR-RFLP, polymerase chain reaction-restricted fragment length polymorphism.

A total of 20 studies in the literature were screened after a preliminary literature search. Among them, nine were eligible for the current meta-analysis. One paper deviating from the HWE was also included because the sensitivity analysis would be performed subsequently (21). Three excluded papers were published by the same author group to the study published by Luo et al. (13), Luo et al. (22), and Du et al. (23). Considering some data was overlapped among these studies, only Xu’s study was included because the sample size in this study was the largest of all of the four studies by the same author group. Five review literature studies were rejected, and three literature studies were excluded because they were not associated with either the *MIF* gene rs755622 G/C polymorphism or CAD (Supplementary Table 2).

### Pooled analyses

A total population of 9 articles and 8,488 participants contributed to those results. The Chinese subgroup reflected 7 articles and 7,583 participants. The Caucasian subgroup reflected 2 articles and 905 participants. In the Caucasian subgroup analysis, Russian, Czech, and Turkish populations were included. Moreover, the Turkish study is a new addition to the previous study by Li et al. (10), and it appeared to change the results significantly in comparison to the findings of Li.

**FIGURE 1:**
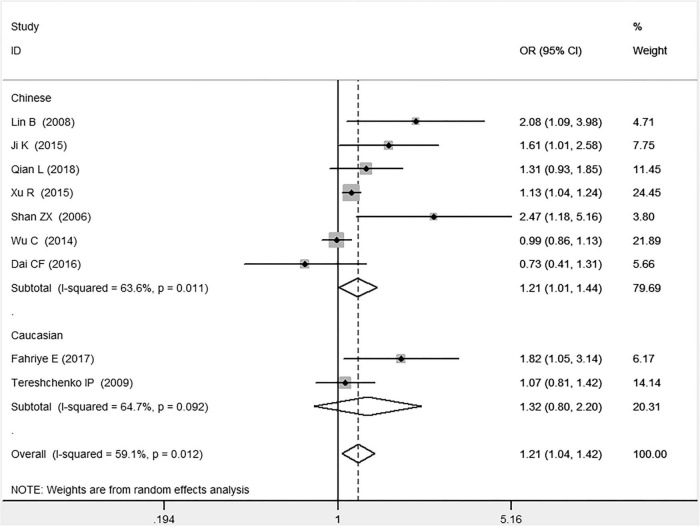
Forest plot of CAD associated with *MIF* gene rs755622 G/C polymorphism under an allelic genetic model (i.e., C allele vs. G allele + C allele of *MIF* gene rs755622 G/C polymorphism).

**FIGURE 2:**
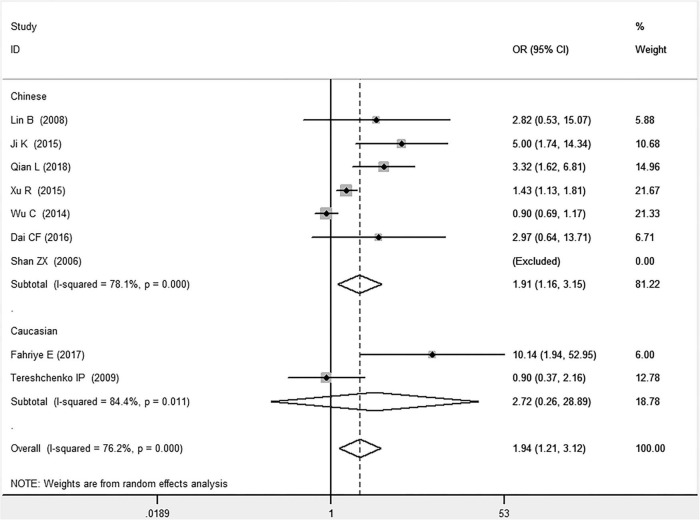
Forest plot of CAD associated with *MIF* gene rs755622 G/C polymorphism under a recessive genetic model (i.e., CC vs. GG + GC of *MIF* gene rs755622 G/C polymorphism).

**FIGURE 3:**
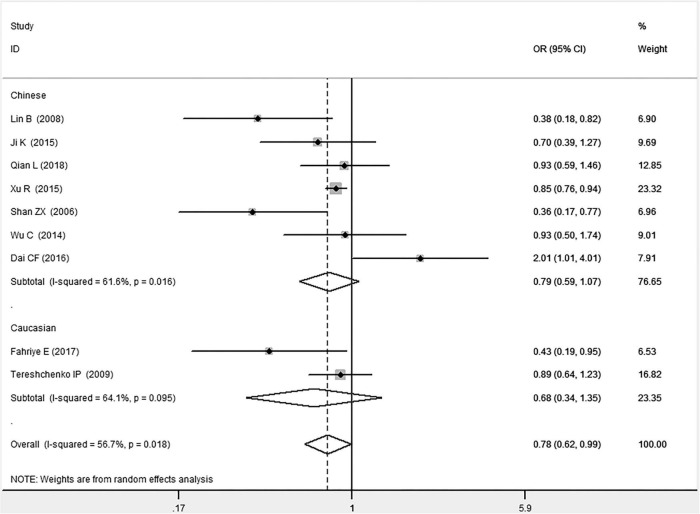
Forest plot of CAD associated with *MIF* gene rs755622 G/C polymorphism under a dominant genetic model (i.e., GG vs. GC + CC of *MIF* gene rs755622 G/C polymorphism).

**FIGURE 4:**
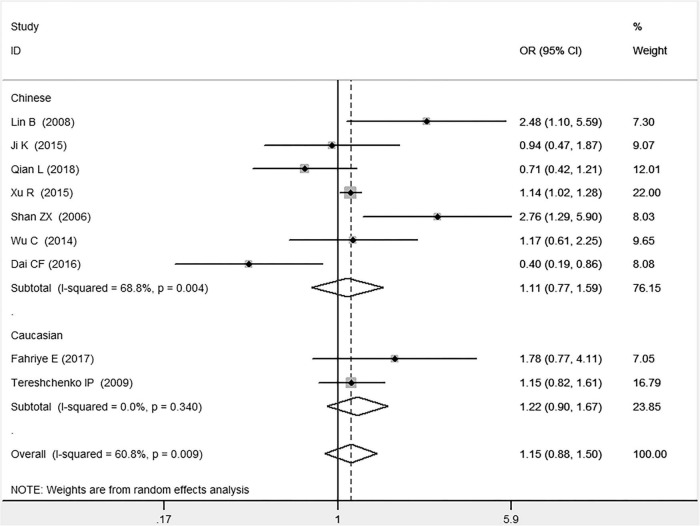
Forest plot of CAD associated with *MIF* gene rs755622 G/C polymorphism under a heterozygous genetic model (i.e., GC vs. GG of *MIF* gene rs755622 G/C polymorphism).

**FIGURE 5:**
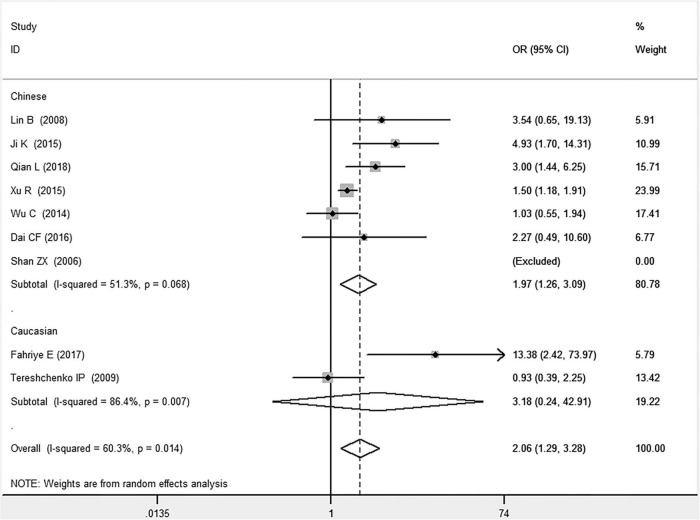
Forest plot of CAD associated with *MIF* gene rs755622 G/C polymorphism under a homozygous genetic model (i.e., CC vs. GG of *MIF* gene rs755622 G/C polymorphism).

**FIGURE 6:**
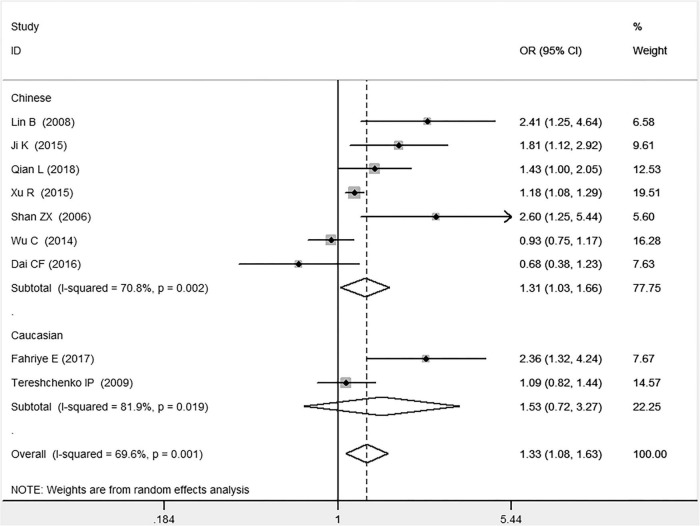
Forest plot of CAD associated with *MIF* gene rs755622 G/C polymorphism under an additive genetic model (i.e., C allele vs. G allele of *MIF* gene rs755622 G/C polymorphism).

In the total population, the *MIF* gene rs755622 G/C polymorphism was significantly associated with CAD under the allelic (OR: 1.213, 95% CI: 1.039–1.417, *P* = 0.014), recessive (OR: 1.945, 95% CI: 1.214–3.115, *P* = 0.006), dominant (OR: 0.781, 95% CI: 0.617–0.989, *P* = 0.041), homozygous (OR: 2.057, 95% CI: 1.289–3.284, *P* = 0.003), and additive (OR: 1.327, 95% CI: 1.081–1.630, *P* = 0.007) genetic models. No significant relation between them was found under the heterozygous (OR: 1.146, 95% CI: 0.818–1.496, *P* = 0.316) genetic model (Table 2 and Figures 1–6).

**TABLE 2:** Meta-analysis results of association between *MIF* gene rs755622 polymorphism and CAD.

Genetic model	Pooled OR (95% CI)	*Z*-value	*P*-value	Literature number	CAD size	Control size	*P*_*heterogeneity*_ (*I*^2^%)
Allelic genetic model	1.213 (1.039–1.417)	2.45	0.014*	9	3,843	4,645	0.012* (59.1%)
Chinese subgroup	1.207 (1.009–1.443)	2.06	0.039*	7	3,349	4,234	0.011* (63.6%)
Caucasian subgroup	1.323 (0.797–2.195)	1.08	0.279	2	494	411	0.092 (64.7%)
Recessive genetic model	1.945 (1.214–3.115)	2.77	0.006*	9	3,843	4,645	<0.0001* (76.2%)
Chinese subgroup	1.912 (1.160–3.152)	2.54	0.011*	7	3,349	4,234	<0.0001* (78.1%)
Caucasian subgroup	2.719 (0.256–28.891)	0.83	0.407	2	494	411	0.011* (84.4%)
Dominant genetic model	0.781 (0.617–0.989)	2.05	0.041*	9	3,843	4,645	0.018* (56.7%)
Chinese subgroup	0.793 (0.586–1.073)	1.51	0.132	7	3,349	4,234	0.016* (61.6%)
Caucasian subgroup	0.676 (0.339–1.349)	1.11	0.267	2	494	411	0.095 (64.1%)
Heterozygous genetic model	1.146 (0.818–1.496)	1.00	0.316	9	3,843	4,645	0.009* (60.8%)
Chinese subgroup	1.106 (0.768–1.592)	0.54	0.588	7	3,349	4,234	0.004* (68.8%)
Caucasian subgroup	1.224 (0.896–1.672)	1.27	0.205	2	494	411	0.340 (0%)
Homozygous genetic model	2.057 (1.289–3.284)	3.02	0.003*	9	3,843	4,645	0.014* (60.3%)
Chinese subgroup	1.971 (1.259–3.086)	2.97	0.003*	7	3,349	4,234	0.068 (51.3%)
Caucasian subgroup	3.183 (0.236–42.908)	0.87	0383	2	494	411	0.007* (86.4%)
Additive genetic model	1.327 (1.081–1.630)	2.71	0.007*	9	3,843	4,645	0.001* (69.6%)
Chinese subgroup	1.306 (1.030–1.657)	2.20	0.028*	7	3,349	4,234	0.002* (70.8%)
Caucasian subgroup	1.534 (0.719–3.272)	1.11	0.269	2	494	411	0.019* (81.9%)

*P < 0.05. MIF, macrophage migration inhibitory factor; CAD, coronary artery disease; CI, confidence interval; OR, odds ratio; CAD size, the total number of CAD cases; control size, the total number of control group; allelic genetic model, C allele vs. G allele + C allele; recessive genetic model, CC vs. GG + GC; dominant genetic model, GG vs. GC + CC; heterozygous genetic model, GC vs. GG, homozygous genetic model, CC vs. GG; additive genetic model, C allele vs. G allele.

In the Chinese subgroup, a significant association between *MIF* gene rs755622 G/C polymorphism and CAD was observed under the allelic (OR: 1.207, 95% CI: 1.009–1.443, *P* = 0.039), recessive (OR: 1.912, 95% CI: 1.160–3.152, *P* = 0.011), homozygous (OR: 1.971, 95% CI: 1.259–3.086, *P* = 0.003), and additive (OR: 1.306, 95% CI: 1.030–1.657, *P* = 0.028) genetic models. There was no significant association between them under the dominant (OR: 0.793, 95% CI: 0.586–1.073, *P* = 0.132) or heterozygous (OR: 1.106, 95% CI: 0.768–1.592, *P* = 0.588) genetic models.

In the Caucasian subgroup, no significant association between them was observed under the allelic (OR: 1.323, 95% CI: 0.797–2.195, *P* = 0.279), recessive (OR: 2.719, 95% CI: 0.256–28.891, *P* = 0.407), dominant (OR: 0.676, 95% CI: 0.339–1.349, *P* = 0.267), heterozygous (OR: 1.224, 95% CI: 0.896–1.672, *P* = 0.205), homozygous (OR: 3.183, 95% CI: 0.236–42.908, *P* = 0.383), or additive (OR: 1.534, 95% CI: 0.719–3.272, *P* = 0.269) genetic models.

As there was significant heterogeneity under all of the genetic models (*P*_*heterogeneity*_ < 0.05), the random-effects models were utilized. After the population was stratified according to ethnicity, the heterogeneity was reduced in the subgroups under the heterozygous and the homozygous genetic models. Under the heterozygous genetic model, the *I*^2^ was reduced from 60.8% in the total population to 0 in the Caucasian subgroup. Under the homozygous genetic model, the *I*^2^ was reduced from 60.3% in the total population to 51.3% in the Chinese subgroup. It was indicated that the different ethnicity was associated with the heterogeneity source. In general, if *P*_*heterogeneity*_ is large, *I*^2^ must be small. The smaller *P*_*heterogeneity*_ is, the greater the heterogeneity is, and *I*^2^ represents how big it is. In Table 2, under the allelic, recessive, dominant, and additive genetic models, it was found that the *P*_*heterogeneity*_ value has the same change trend with the *I*^2^ in the Caucasian subgroup. In the heterogeneity test, the results of the *Q*-test are inconsistent with those of the H and *I*^2^-test, which is due to the small number of studies in the Caucasian subgroup, and the *Q*-test and interval range estimation are often inaccurate (24). *Q*-value changes with the change in the number of studies. H and *I*^2^-test statistics are often used to correct the influence of the study’s number on the *Q*-value. Their value would not change with the study’s number, and the heterogeneity test results are more robust and reliable.

### Publication bias diagnostics

**FIGURE 7:**
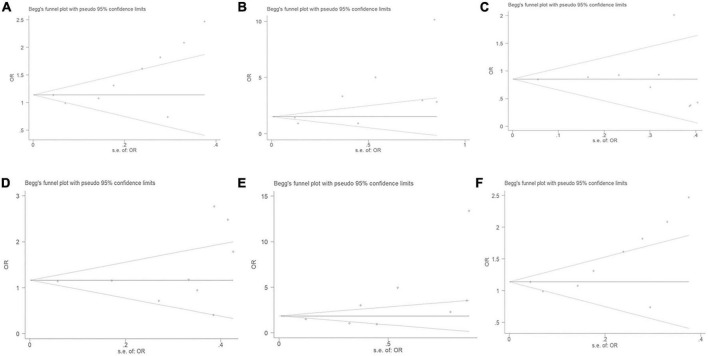
The Begg’s funnel plot for studies of the association of CAD and *MIF* gene rs755622 G/C polymorphism. **(A)** The Begg’s funnel plot for studies of the association under an allelic genetic model (i.e., C allele vs. G allele + C allele of *MIF* gene rs755622 G/C polymorphism). **(B)** The Begg’s funnel plot for studies of the association under a recessive genetic model (i.e., CC vs. GG + GC of *MIF* gene rs755622 G/C polymorphism). **(C)** The Begg’s funnel plot for studies of the association under a dominant genetic model (i.e., GG vs. GC + CC of *MIF* gene rs755622 G/C polymorphism). **(D)** The Begg’s funnel plot for studies of the association under a heterozygous genetic model (i.e., GC vs. GG of *MIF* gene rs755622 G/C polymorphism). **(E)** The Begg’s funnel plot for studies of the association under a homozygous genetic model (i.e., CC vs. GG of *MIF* gene rs755622 G/C polymorphism). **(F)** The Begg’s funnel plot for studies of the association under an additive genetic model (i.e., C allele vs. G allele of *MIF* gene rs755622 G/C polymorphism). The horizontal and vertical axes correspond to the OR on log scale and SE(logOR), respectively. OR, odds ratio; SE, standard error.

No significant publication bias was observed through the Begg’s test under the allelic (*T* = 2.18, *P* = 0.066), dominant (*T* = –0.04, *P* = 0.973), heterozygous (*T* = 0.87, *P* = 0.414), homozygous (*T* = 1.89, *P* = 0.107), or additive genetic models (*T* = 1.93, *P* = 0.095) (Figure 7). It was suggested that there was no significant publication bias in the current meta-analysis under these genetic models. A significant publication bias was only observed under the recessive genetic models (*T* = 2.59, *P* = 0.041).

## Sensitivity analysis

**FIGURE 8:**
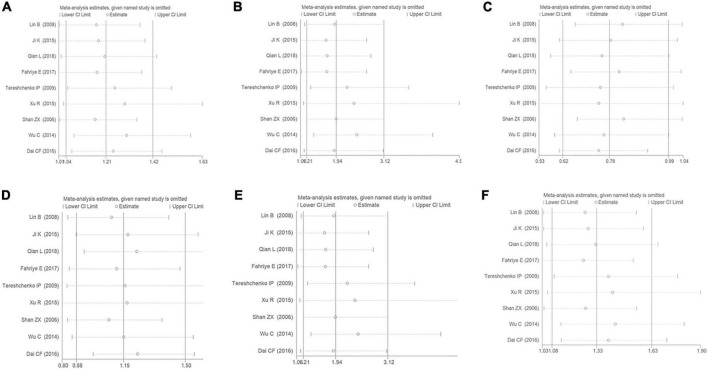
The sensitivity analysis on the association of CAD and *MIF* gene rs755622 G/C polymorphism. **(A)** The sensitivity analysis on the association under an allelic genetic model (i.e., C allele vs. G allele + C allele of *MIF* gene rs755622 G/C polymorphism). **(B)** The sensitivity analysis on the association under a recessive genetic model (i.e., CC vs. GG + GC of *MIF* gene rs755622 G/C polymorphism). **(C)** The sensitivity analysis on the association under a dominant genetic model (i.e., GG vs. GC + CC of *MIF* gene rs755622 G/C polymorphism). **(D)** The sensitivity analysis on the association under a heterozygous genetic model (i.e., GC vs. GG of *MIF* gene rs755622 G/C polymorphism). **(E)** The sensitivity analysis on the association under a homozygous genetic model (i.e., CC vs. GG of *MIF* gene rs755622 G/C polymorphism). **(F)** The sensitivity analysis on the association under an additive genetic model (i.e., C allele vs. G allele of *MIF* gene rs755622 G/C polymorphism).

The sensitivity analysis has been performed under the six genetic models to analyze the results’ stability. It was observed that even if any of the individual studies were eliminated from this meta-analysis, the primary outcome was not influenced. It was suggested that the present meta-analysis’s results were considerably stable (Figure 8).

## Discussion

In the current meta-analysis, the *MIF* gene rs755622 G/C polymorphism is found to be remarkably associated with the development of CAD under allelic (OR: 1.213), recessive (OR: 1.945), dominant (OR: 0.781), homozygous (OR: 2.057), and additive (OR: 1.327) genetic models. The C allele of the *MIF* gene –173G/C polymorphism may increase the CAD risk. The significant association between *MIF* gene rs755622 G/C polymorphism and CAD is found in the Chinese subgroup (*P* < 0.05) but not in the Caucasian subgroup (*P* > 0.05). Given that only two individual studies are included in the Caucasian subgroup, the result in this subgroup needs to be confirmed in more studies in the future. Only the random-effects models are used because of the significant heterogeneity among individual studies. Heterogeneity is distinctly reduced in the subgroup analysis under heterozygous and homozygous genetic models. The heterogeneity source is suggested to be related to different ethnicities.

CAD is a complex multifactorial disease resulting from the interaction of genetic and environmental factors. CAD is common in the elderly, and the formation of atherosclerotic plaque in the coronary artery is the main cause of CAD. Atherosclerosis is a disease of fat deposition, and inflammation plays a key role in the formation and development of atherosclerotic thrombosis. Many inflammatory factors, chemokines, and enzymes are involved in the pathogenesis of atherosclerosis (25).

MIF was discovered by Bloom and Bennett in a hypersensitivity experiment on guinea pigs in 1966 and was named for its ability to inhibit macrophage mobility (26). Later studies found that MIF can participate in a series of stress responses of the host to microbial infection; activate macrophages; inhibit migration; and enhance adhesion, phagocytosis, and tumor-killing activity (27–29). MIF is widely expressed in T cells, mononuclear macrophages, various hematopoietic cells, central and peripheral nerve cells, renal tubular epithelial cells, fat cells, vascular endothelial cells, liver cells, lung, prostate, and testis. MIF may be involved in various physiological and pathological processes of the body, including inflammatory response, immune response, tumor genesis, and tissue damage and repair (30).

When atherosclerotic plaques occur, serum MIF levels increase rapidly, suggesting that MIF may be involved in the occurrence of atherosclerosis. MIF can be rapidly released from macrophages, cardiomyocytes, and vascular endothelial cells under the stimulation of harmful factors (31). The released MIF can promote the transformation of macrophages into foam cells and accelerate the progression of atherosclerosis (32). As an important proinflammatory factor and chemokine, MIF can recruit macrophages, T lymphocytes, and other inflammatory immune cells to participate in the inflammatory response in atherosclerotic plaque (33, 34) and stimulate macrophages and lymphocytes to secrete inflammatory factors, including interleukin-6, interleukin-8, tumor necrosis factor-α, and intracellular adhesion factor. MIF can remarkably improve the level of inflammatory response in atherosclerotic plaques (35). Hence, considering the current research results, MIF may be a biomarker for the early diagnosis of CAD and a new target for the treatment of CAD, especially in the Chinese population.

*MIF* gene has multiple polymorphic loci, including –173G/C, 254T/C, and 656C/G. Among these polymorphic loci, –173G/C polymorphism is closely related to CAD. Given that the *MIF* gene –173 C allele has an AP4 response element-binding site, the difference in the mononucleotide may result in different affinities with the AP4 response element, thus affecting the MIF expression level. Donn et al. found that healthy volunteers with the –173C genotype (GC + CC) have significantly higher serum MIF levels than those with the –173GG genotype (36). Hence, the *MIF* gene –173G/C mutation can lead to a high MIF level and promote CAD development, which is in line with the outcome of the present meta-analysis.

In the meta-analysis by Li et al. (10), OR values are larger under the C/G, CC/GG, and CC/CG + GG genetic models than the results obtained from the current meta-analysis. Under the CG/CC and GG/CG + CC genetic models, the OR values in their meta-analysis are smaller than the current result. Although similar methodological techniques are used in Li’s paper, the current work has added value. A total of 8,488 participants analyzed herein compared with the 2,736 participants evaluated in Li mitigate that concern of value-added. In addition, five of six individual studies in Li’s meta-analysis are also included herein. The study of Luo et al. (13) is excluded due to replication of the study by Xu (12), which is included in the current research. In another meta-analysis by Lian et al. (11), two individual studies published by a similar author group are included in their meta-analysis (12, 13). Despite the similar method used and similar effect size obtained in their meta-analysis and the current research, overlapping data may affect the results.

Limitations are present in this study. The sample size is relatively small. The data on the Caucasian population are especially limited. More studies from a large sample size of Caucasian populations are needed to cover the shortage. In addition, the environmental factors of hyperlipidemia, hypertension, and diabetes also have significant effects on CAD susceptibility. Other gene polymorphisms, e.g., *PCSK9* gene E670G polymorphism (37), *ALDH2* gene G487A polymorphism (38), *CDKN2B-AS1* gene rs4977574 A/G polymorphism (39), and *FVII* gene R353Q polymorphism (40), may also be involved with CAD susceptibility.

The *MIF* gene –173C/G polymorphism is associated with CAD risk. The C allele of the *MIF* gene –173C/G polymorphism may be the risk factor for CAD, especially in the Chinese population. More studies on the relationship between *MIF* gene –173C/G polymorphism and CAD need to be conducted to further verify this conclusion. *MIF* gene expression studies are necessary for confirmation.

## Data availability statement

The original contributions presented in this study are included in the article/Supplementary material, further inquiries can be directed to the corresponding author/s.

## Author contributions

Y-YL and HW researched the data. Y-YL wrote the manuscript, researched the data, contributed to the discussion, and reviewed/edited the manuscript. Y-YL and Y-YZ reviewed/edited the manuscript. All authors contributed to the article and approved the submitted version.

## Supplementary material

Supplementary Table 1 PRISMA checklist.

Supplementary Table 2 PRISMA flow diagram.
