# Supplementary material for: Macrophage migration inhibitory factor gene rs755622 G/C polymorphism and coronary artery disease: A meta-analysis of 8,488 participants
Source: Front Cardiovasc Med. 2022 Sep 14;9:959028. doi: 10.3389/fcvm.2022.959028 (PMC9515403; doi:10.3389/fcvm.2022.959028)
Supplement: Supplementary Table 2 — PRISMA flow diagram. [file Table_2.docx]

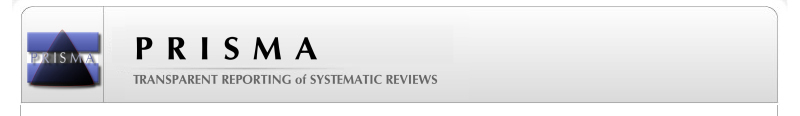
**PRISMA 2009 Flow Diagram**

Records excludedfor duplicate publication (n=3)

Records excluded for no association with *MIF* gene rs755622 G/C polymorphism or CAD

(n =3)

Full-text articles excluded for deviation from HWE(n=0)

Records excludedfor review characteristic
(n=5)

Studies included in qualitative synthesis
(n =9)

Full-text articles assessed for eligibility
(n =12)

Records screened
(n =12)

Records after duplicates removed
(n =17)

Additional records identified through other sources
(n =0)

## Identification

## Eligibility

## Included

## Screening

Records identified through database searching
(n =20)
